# Is Decline Rate of Intact Parathyroid Hormone Level a Reliable Criterion for Early Discharge of Patients after Total Thyroidectomy?

**Published:** 2017-09

**Authors:** Mohsen Kolahdouzan, Shahab Shahabi Shahmiri, Seyed Mozafar Hashemi, Behrouz Keleidari, Masoud Nazem, Rastin Mohammadi Mofrad

**Affiliations:** 1 *Department of Surgery, Faculty of Medicine, Isfahan University of Medical Sciences, Isfahan, Iran. *

**Keywords:** Hypocalcemia, Parathyroid hormone, Thyroidectomy

## Abstract

**Introduction:**

Parathyroid dysfunction leading to symptomatic hypocalcemia is not uncommon following a total thyroidectomy and is often associated with significant patient morbidity and a prolonged hospital stay. The current study aimed at evaluating the comparative predictive role of serum calcium and intact parathyroid hormone (iPTH) for post-thyroidectomy hypocalcemia.

**Materials and Methods:**

This prospective study was performed in 83 consecutive patients undergoing total thyroidectomy. Laboratory data such as serum calcium, vitamin D level, serum iPTH and serum phosphorus levels before surgery, postoperative calcium, and PTH levels measured after 1 and 6 hours and on the first postoperative day (1POD) were recorded.

**Results:**

Among the 83 patients, the mean (SD) age was 45.87 (12.57) years (range, 21–72 years); 70 (84.3%) patients were female. Final pathology was benign for 47 (56.6%) patients and malignant for 36 (43.4%) patients. In total, lymph node dissections were performed in 19 subjects (22.9%). On histological examination of the specimens, the parathyroid gland was found to have been removed inadvertently in 13 (15.7%) cases. In total, 35 (40.9%) patients developed hypocalcemia after thyroidectomy; receiver operating characteristic (ROC) analysis showed that a cut-off value of 15.39 pg/ml for iPTH, with a decline rate of 73% 1 hour after thyroidectomy is a significant predictor of hypocalcemia (area under the curve [AUC], 0.878; 95% confidence interval [CI], 0.79–0.96, P<0.0001) compared with calcium <8 mg/dl (2 mmol/L) with AUC=0.639; 95% CI, 0.51–0.76); P=0.067).

**Conclusion:**

The current study showed that the decline rate in iPTH is a more reliable factor for hypocalcemia after total thyroidectomy than serum calcium. Patients with a decline rate <73% in iPTH could be discharged at 1POD without supplementation.

## Introduction

Total thyroidectomy has evolved as the treatment of choice for most malignant and many benign thyroid disorders. Hypocalcemia as a result of hypoparathyroidism is the most common complication of thyroidectomy. Although improvements in surgical technique have made thyroidectomy a safe operation, transient hypoparathyroidism is seen after 0.3–49% of thyroidectomies, while permanent hypoparathyroidism is less likely and has been reported in up to 13% of cases (1–4). This can be a result of manipulation of the parathyroid gland during surgery, devascularization of the parathyroid gland, or from inadvertent removal of the parathyroid gland with the thyroid specimen (5–8). Analysis of the relative decrease between preoperative and postoperative intact parathyroid hormone (iPTH) levels ([preoperative iPTH − postoperative iPTH]/ preoperative iPTH) should increase diagnostic precision.

Currently, the emphasis is on the early discharge of thyroidectomy patients. Some surgeons prescribe calcium supplementation routinely after thyroidectomy to prevent symptoms of hypocalcemia, whereas others rely upon postoperative serum calcium levels. Calcium levels can decrease up to 5 days after the thyroidectomy. Serum calcium level on the first postoperative day has a lack of sensitivity and can lead to false-negative results (9,10). In order to obtain an earlier diagnosis of hypocalcemia, a decrease in calcium levels has been evaluated; however, no correlation has been established between this decrease and the presence of postoperative hypocalcemia. Other authors have highlighted the relevance of serum levels of iPTH (11–14).

Postoperative iPTH and calcium levels, as well as clinical factors such as lymph node dissection, auto-transplantation, and indication for thyroidectomy, have been studied in order to anticipate the need for calcium supplementation and to predict the development of hypocalcemia symptoms. Biochemical studies of post-thyroidectomy patients have demonstrated that PTH levels at various times correlate with the development of symptomatic hypocalcemia, although the optimal timing remains unclear. On the other hand, vitamin D deficiency is common in Iran and therefore assumes importance as a factor that could influence post-thyroidectomy management. However, it remains unclear whether low vitamin D level is a risk factor for the development of post-thyroidectomy hypocalcemia (15,16). The current study aimed at evaluating the comparative predictive role of serum calcium and iPTH drawn at several time points; 1 and 6 hours postoperatively and on postoperative Day 1 (POD1) for hypocalcemia. As the few studies evaluating the diagnostic value of the decline in iPTH levels after thyroidectomy for hypocalcemia have reported different results, our study will clarify the efficacy of decline in the value of iPTH for the early diagnosis of hypocalcemia after total thyroidectomy. The results of this study would be helpful in identifying those patients who are not at risk of hypocalcemia for early discharge at POD1 without supplementation.

## Materials and Methods

This prospective observational study was performed in 83 patients who consecutively underwent total thyroidectomy in the general surgery ward of Alzahra Hospital, Isfahan, Iran from April 2015 and January 2016. Patients with concomitant parathyroid disease, renal failure and unilateral thyroid lobectomy were excluded. All operations were performed by one surgeon and senior residents, and consisted of total thyroidectomy. Preservation of the parathyroid gland and its blood supply was attempted in all cases. The study protocol was approved by the local Ethics Committee of Isfahan University of Medical Sciences. Written informed consent was obtained from all participants in our study.

Demographic and clinical data were collected for all patients and included age, gender, histologic diagnosis, pathology results (including notation of any parathyroid tissue discovered in the specimen) and hormonal status. Instances of hypocalcemia were defined as serum calcium less than 8 mg/dl and/or when patients experienced symptoms such as circumoral numbness, tingling of the fingers and toes, paresthesia or muscle irritability or had positive Chvostek’s signs (17). Instances of hypocalcemia were recorded on 1POD as well as at a 2-week postoperative visit. Replacement therapy with oral calcium supplements and vitamin D analog (calcitriol, Rocartrol) was initiated in patients with hypocalcemia. Patients were discharged when the serum calcium level was more than 8 mg/dl and without signs and symptoms of hypocalcemia. Additionally, laboratory data such as serum calcium, vitamin D level, serum iPTH and serum phosphorus levels before surgery, postoperative calcium and iPTH levels measured at 1 and 6 hours post-surgery and on POD1 were recorded. A peripheral blood sample was obtained 1 hour after surgery and in the recovery room, 6 h after surgery and on the morning after surgery

Data were analyzed using SPSS (Version 15.0 SPSS Inc., Chicago, IL, USA). Quantitative data were expressed as mean±standard deviation (SD), while qualitative data were expressed as frequency (percentage). Categorical variables and categorical data were compared between groups using the Chi-square test, while normality distributed quantitative data were analyzed using the independent samples t-test. Receiver operating characteristic (ROC) curve analysis was conducted to evaluate the predictive roles of iPTH and calcium for post-thyroidectomy hypocalcemia. Area under the cure (AUC) and 95% confidence interval (CI), sensitivity and specificity, negative and positive predicative and overall accuracy values were reported. A two-sided P<0.05 was considered to be statistically significant.

## Results

**Fig 1:**
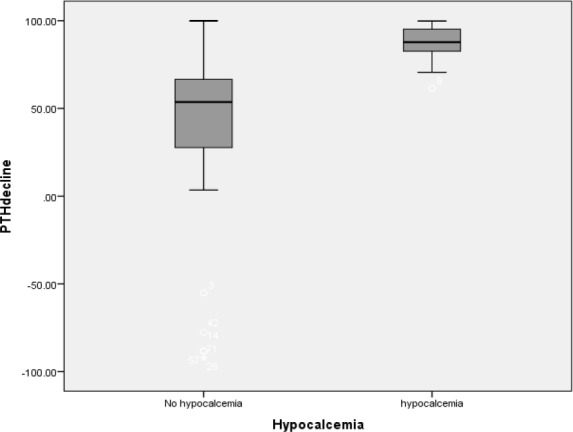
Comparison of postoperative iPTH levels obtained immediately after thyroidectomy between patients with and without hypocalcemia

During the study period from April 2015 to January 2016, 83 patients underwent total thyroidectomy with or without neck dissection in our hospital by one surgeon. Among the studied patients, 70 (84.3%) were female with a mean (SD) age of 45.87 (12.57) years (range, 21–72 years). Final pathology was benign for 47 (56.6%) patients. In total, lymph node dissection was performed in 19 (22.9%) subjects. Based on histological examination of the specimens, unintentional parathyroid gland excision was found in 13 (15.7%) patients (one parathyroid gland in 11 cases and two glands in two cases). In total, symptomatic hypocalcemia developed in 34 (40.9%) patients after surgery; of whom seven (20.5%) were admitted to the hospital for intravenous calcium supplement. The results are summarized in Table 1. As shown, the mean (SD) value of serum iPTH level 1 hour post-thyroidectomy was 7.78 (6.75) pg/ml and 27.97 (6.18) pg/ml, in patients with and without hypocalcemia, respectively (P<0.001) (Fig.1). Mean (SD) PTH levels were significantly more reduced in patients who developed hypocalcemia than in those without hypocalcemia (87.83% (20) vs. 36.67% (51.82); P<0.0001). Mean (SD) serum calcium concentration 6 hours after thyroidectomy in patients with and without hypocalcemia was 8.60 (0.20) mg/dL and 8.78(0.39) mg/dL, respectively (P=0.07). No significant differences were found between patients with and without hypocalcemia in terms of sex, age, type of pathology (benign or malignant), vitamin D deficiency status or lymph node dissection (Table. 1).

**Table 1:** Demographic, operative, pathologic and laboratory characteristics of study patients

**Variable**	**All patients**	**With hypocalcemia**	**Without hypocalcemia**	**P-value**
Sex				
Male, n (%) Female, n (%)	13 (15.7) 70 (84.3)	6 (46.1) 28 (40)	7 (53.8) 42 (60)	0.452
Age, mean (SD)	45.87 (12.57)	45.38 (12.44)	46.20 (12.78)	0.771
Pathological finding				
Benign, n (%) Malignant, n (%)	47 (56.6) 36 (43.3)	23 (49) 11 (30.5)	24 (51) 25 (69.5)	0.071
Neck dissection				
Yes, n (%) No, n (%)	19 (22.8) 64 (77.1)	7 (36.8) 27 (42.1)	12 (63.1) 37 (57.8)	0.444
Parathyroid glands				
Seen, n (%) Not seen, n (%)	13 (15.7) 70 (84.3)	(38.4) 29 (41.4)	8 (61.5) 41 (58.5)	0.548
Mean (SD) preoperative PTH level, pg/ml	58.20 (26.31)	28.03 (4.80)	24.93 (3.56)	0.232
Mean (SD) postoperative PTH level, pg/ml (1 hour)	19.70 (16.47)	7.78 (6.75)	27.97 (16.18)	0.001
Mean (SD) % PTH decline	57.63 (47.40)	87.83 (9.20)	36.67 (51.82)	0.001
Mean (SD) postoperative calcium level (6 hours) mg/dl	8.67 (0.33)	8.60 (0.20)	8.72 (0.39)	0.07
Mean (SD) postoperative calcium level (POD1) mg/dl	8.32 (0.32)	8.22 (0.20)	8.38 (0.39)	0.026
Mean (SD) serum vitamin D, ng/ml	26.16 (17.65)	27.57 (14.87)	25.20 (19.48)	0.617
Vitamin D deficiency, n (%)	28 (33.7)	8 (23)	20 (40)	0.16

**Fig 2:**
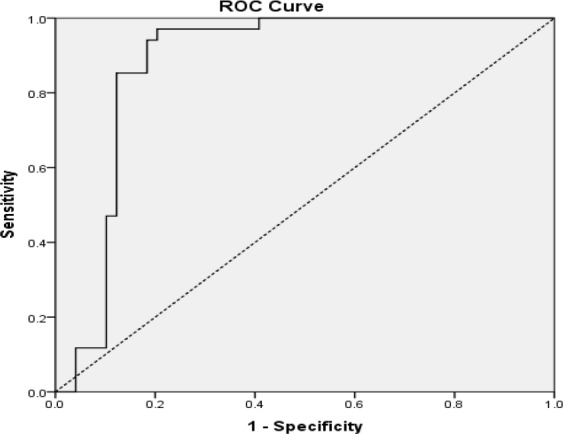
ROC analysis of decline in PTH in predicting post-thyroidectomy hypocalcemia (AUC, 0.878; 95% CI, 0.79–0.96, P<0.001).

**Fig 3:**
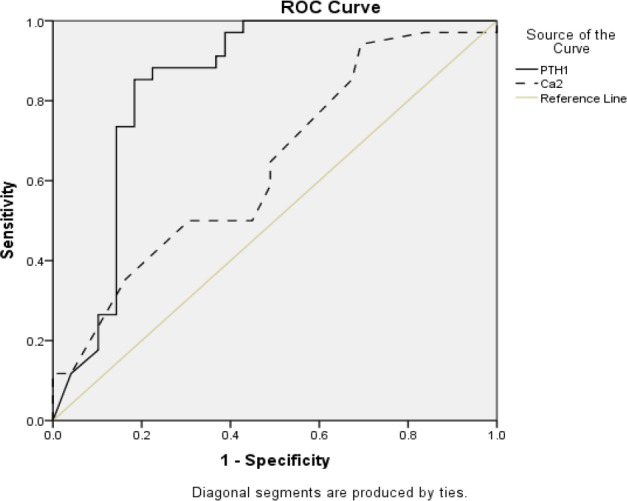
ROC analysis of PTH level 1-hour post-thyroidectomy and serum calcium concentration POD1 in predicting post-thyroidectomy hypocalcemia (AUC, 0.843; 95% CI, 0.75–0.93 for PTH and AUC, 0.639; 95% CI, 0.51–0.76 for POD1 calcium

The cut-off value of 15.39 pg/ml for iPTH could be considered a reliable value for predicting hypocalcemia (AUC, 0.843; 95% CI, 0.75–0.93; P<0.0001) with sensitivity, specificity, positive and negative predictive values of 88.23%, 77.55%, 74% and 90%, respectively (overall accuracy, 81.92%). An evaluation of the diagnostic values of iPTH decline rate based on an ROC analysis showed that a 73% decline in iPTH level 1 hour after thyroidectomy is a significant cut-off value for occurrence of hypocalcemia (AUC, 0.878; 95% CI, 0.79–0.96; P<0.0001) (Fig.2); sensitivity, 94%; specificity, 81.63%; positive predictive values 78%; negative predictive values, 95.23%; and overall accuracy, 86.74%. As can be seen in Figure 3, iPTH levels have a significantly higher predictive role than POD1 calcium (P<0.0001). Sensitivity, specificity, positive and negative predictive values for POD1 calcium <8 mg/dl (2 mmol/L) were 35%, 83%, 60% and 65%, respectively (overall accuracy, 63.8%) (AUC, 0.639; 95% CI, 0.51–0.76); P=0.067).

Vitamin D deficiency (defined as vitamin D<20 ng/ml) was seen in 28 (33.7%) patients. The results of our study show that vitamin D status was not associated with the development of post-thyroidectomy hypocalcemia (P=0.16). Mean (SD) postoperative PTH in patients with and without vitamin D deficiency were 23.49 (17.96) and 17.77 (15.48), respectively (P=0.13). No significant difference was observed between patients with and without vitamin D deficiency in terms of decline in iPTH levels for predicting development of hypocalcemia (AUC, 0.888 and 0.830 for patients with and without vitamin D deficiency, respectively [P=0.32]).

## Discussion

Thyroidectomy is evolving into an outpatient surgery, although the presence of transient hypocalcemia postoperatively lengthens the duration of hospital stay. Therefore, there is increased interest in the identification of risk factors for the development of post-thyroidectomy hypocalcemia. Over the last several years, a few studies have been conducted using measurement of iPTH within minutes or hours after thyroidectomy in an attempt to predict hypocalcemia. Trung and colleagues concluded a postoperative PTH level <12 pg/ml drawn after 1 hour accurately correlated with the development of significant hypocalcemia, with a positive predictive value of 90% and a negative predictive value of 98% (18). In a 2015 study conducted by Selberherr in 237 patients, it was found that measurement of iPTH on the morning after surgery allows accurate prediction of postoperative parathyroid function in 99% of cases (19). Results of this study showed that sensitivity, specificity, and positive and negative predictive values for iPTH were 88.23%, 77.55%, 74% and 90%, respectively, with an overall accuracy of 81.92% (AUC, 0.843; 95% CI, 0.75–0.93; P=0.000).

The researchers in the present study have raised the iPTH cut-off level from 12 to 15.39 pg/ml in the development of a prediction for a single 1-hour PTH level in predicting the risk of development of hypocalcemia. However, the use of an absolute value for postoperative iPTH has been controversial because of a lack of sensitivity or specificity achieved, depending on the threshold considered. Analysis of the relative decrease between preoperative and postoperative iPTH levels ([preoperative iPTH − postoperative iPTH]/preoperative iPTH) should have increased diagnostic precision. However, there is no consensus for the decrease in thresholds, the type of measurement or the timing of the measurement for iPTH.

In 2012, Lecerf and colleagues concluded that patients with a decrease in iPTH less than 68.5% can be discharged on postoperative Day 1 without any supplementation. The overall accuracy was 96.4% (95% CI, 91.7–98) (20). In a 2015 study by Puzziello et al. in 75 patients, it was concluded that hypercalcemic patients on the second postoperative day had a 62% relative decrease in iPTH 2 hours after thyroidectomy. The ROC curve for the iPTH percentage showed an excellent accuracy for predicting total hypocalcemia (AUC, 0.93) compared with absolute decrease in iPTH. Equally noteworthy is that relative iPTH >62% had a 100% sensitivity and specificity for predicting hypocalcemia (21).

The results of the present study suggest the relevance of perioperative iPTH decline, which is more precise than iPTH level alone in the early diagnosis of hypocalcemia after total thyroidectomy. A threshold of iPTH decline of 73% has a sensitivity of 94%, specificity of 81.63%, positive predictive value of 78%, negative predictive value of 95.23% and overall accuracy of 86.74% (AUC, 0.878; 95% CI, 0.79–0.96; P<0.001).

Vitamin D deficiency has been claimed to influence postoperative hypocalcemia and interfere with the accuracy of PTH prediction, ostensibly by causing secondary hyperparathyroidism and false elevation of PTH. Vitamin D deficiency is highly prevalent in Iran and therefore a cause for concern. This was reconfirmed in 28 out of 83 patients (33.7%) in the present study too. A positive role of vitamin D deficiency in the development of post-thyroidectomy hypocalcemia has been proposed by some researchers (22–24). They hypothesize that, in patients with parathyroid ischemia/injury, the parathyroid gland is unable to secrete PTH and hence the maintenance of calcium homeostasis is dependent on the vitamin D level, as vitamin D increases the intestinal calcium absorption. In vitamin-D-deficient patients, this mechanism of increased calcium absorption is impaired; hence, these patients are prone to developing hypocalcemia (24,25).

Similar to Jacoob and colleagues study in 2016 (26), it was revealed in this study that vitamin D status did not increase the risk of post-thyroidectomy hypocalcemia, nor did it interfere with the predictability of PTH as a marker of post-thyroidectomy hypocalcemia (27-30). Further, Nhan et al. reported a protective effect of vitamin D deficiency for hypocalcemia. They suggest that the secondary hyperparathyroidism caused by vitamin D deficiency results in the ability of the parathyroid gland to secrete more PTH and thus protect against the development of postoperative hypocalcemia (20).

The occurrence of hypocalcemia after total thyroidectomy is predominantly due to trauma/ischemia to the parathyroid gland resulting in hypoparathyroidism. PTH regulates 1-a-hydroxylase in the kidney, which is the enzyme that converts 25-hydroxy-vitamin D to its biologically active form, 1,25-dihydroxy-vitamin D. Thus, an explanation for the lack of association between vitamin D deficiency and post-thyroidectomy hypocalcemia is that hypoparathyroidism results in decreased conversion of 25-hydroxy-vitamin D to 1,25-dihydroxy-vitamin D, regardless of the amount of 25-hydroxy-vitamin D available.

We report a similar finding with a postoperative PTH cut-off of <15 pg/ml. When comparing the area under the ROC curve for both groups, a small increase is seen in the value of AUC in the group with vitamin D deficiency as compared with group without vitamin D deficiency (0.888 vs. 0.830), but this was not significant (P=0.32). Hence, vitamin D deficiency did not affect the accuracy of postoperative PTH in predicting hypocalcemia in the present study. This is in contrast with the findings of other researchers who have shown that postoperative PTH is not a reliable marker in predicting postoperative hypocalcemia in patients with vitamin D deficiency (22,23). In a study of 203 patients undergoing thyroidectomy, Pradeep et al. reported the presence of secondary hyperparathyroidism in the group with vitamin D deficiency (24). Preoperative PTH in this group (60.35±16.06) pg/ml was significantly higher than that in the vitamin D-sufficient group (22.4±7.12 pg/ml ; p=0.0001). The authors also reported a higher postoperative PTH in the vitamin-D-deficient group (16±9.77) compared with the vitamin D-sufficient group (7.13±1.79) among hypercalcemic patients. They concluded that secondary hyperparathyroidism influences the postoperative PTH level in vitamin-D-deficient patients, and hence it is not a reliable predictor for hypocalcemia in this subset of patients. In the present study, the mean preoperative PTH was similar in both the groups (58.85±24.98 in patients with vitamin D deficiency and 56.93±29.18 in the group without vitamin D deficiency; P=0.76). Therefore, our conclusion that the accuracy of postoperative PTH to predict post-thyroidectomy hypocalcemia is not affected by vitamin D deficiency may be attributed to the absence of secondary hyperparathyroidism in this study. Thus, we recommended that the use of relative decrease between preoperative and postoperative iPTH levels ([preoperative iPTH − postoperative iPTH]/preoperative iPTH) in vitamin-D-deficient patients should have increased predictive values.

Additionally, a mild decrease in serum calcium levels was observed in all patients regardless of the eventual development of persistent hypocalcemia. It was actually found that postoperative serum calcium on Day 1 did not consistently correlate with symptomatic hypocalcemia and was not a reliable predictor alone, with sensitivity, specificity, and positive and negative predictive values for POD1 calcium less than 8 mg/dl (2 mmol/L) being 35%, 83%, 60% and 65%, respectively (overall accuracy, 63.8%) (AUC, 0.639; 95% CI, 0.51–0.76; P=0.067).

Central neck dissection could contribute to hypoparathyroidism via disruption of the feeding arteries of the parathyroid glands and ischemic injury. Therefore, preservation of the vascular supply of the parathyroid glands is more important in the prevention of postoperative hypoparathyroidism.* En bloc* resection of thyroid cancer with central compartment node dissection could increase the risk of injury to the blood supply of the parathyroid gland. We have performed central lymph node dissection after completion of thyroidectomy without any dissection lateral to the parathyroid glands. In this way, the blood flow of the final branch to the parathyroid gland is preserved. In contrast with some previous studies, the subgroup analysis in the present study demonstrated that neither the indication of thyroidectomy (benign or malignant), nor lymph node dissection, nor the presence of the parathyroid gland in pathology were a significant risk factors for symptomatic hypocalcemia in our study (as shown in Table 1). Therefore, it is suggested that these risk factors are evaluated in a larger study in the future (31–33).

A final word is that the majority of patients in the study were vitamin D deficient; however, this did not increase the risk of post-thyroidectomy hypocalcemia, nor did it interfere with the predictability of PTH as a marker of post-thyroidectomy hypocalcemia. Further, relative decline in postoperative iPTH was not shown to be a strong predictor of hypocalcemia.

## Conclusions

Patients with a decrease in iPTH less than 73% can be discharged at postoperative Day 1 without any supplementation. Patients with an iPTH decline greater than 73% should be given calcium and vitamin D supplementation before symptoms appear. The researchers hope these findings will help to lower overall hospital costs by minimizing laboratory draws and facilitating early discharge of low-risk patients.
